# Comparison of Serum and Cervical Mucus Docosahexaenoic Acid (DHA) Levels in Patients with Polycystic Ovary Syndrome and Healthy Controls

**DOI:** 10.3390/jcm14248899

**Published:** 2025-12-16

**Authors:** Cigdem Can Bayrak, Bulent Yilmaz, Mehmet Kagitci, Onur Ince, Sibel Mataraci Karakas, Adnan Yilmaz

**Affiliations:** 1 Department of Gynecology and Obstetrics, Recep Tayyip Erdogan University Faculty of Medicine, Rize 53100, Türkiye; drbulentyilmaz@yahoo.com (B.Y.); mehmet.kagitci@erdogan.edu.tr (M.K.); 2 Department of Gynecology and Obstetrics, Health Ministry Bucak State Hospital, Burdur 15300, Türkiye; 3 Department of Obstetrics and Gynecology, Health Ministry Izmir City Hospital, Izmir 35540, Türkiye; 4 Department of Obstetrics and Gynecology, Hacettepe University Faculty of Medicine, Ankara 06100, Türkiye; onurincemd@gmail.com; 5 Department of Biochemistry, Recep Tayyip Erdogan University Faculty of Medicine, Rize 53100, Türkiye; sibel.karakas@erdogan.edu.tr (S.M.K.); adnan.yilmaz@erdogan.edu.tr (A.Y.)

**Keywords:** polycystic ovary syndrome (PCOS), docosahexaenoic acid (DHA), cervical mucus, fatty acids, omega-3, inflammation, infertility

## Abstract

**Background**: Polycystic ovary syndrome (PCOS) is a prevalent proinflammatory condition. Docosahexaenoic acid (DHA), an omega-3 fatty acid obtained through the diet, is known for its anti-inflammatory properties. This study aimed to compare DHA concentrations in serum and cervical mucus between women with PCOS and healthy controls. **Methods**: This prospective cross-sectional study included 42 women with PCOS and 42 healthy controls aged 18–40 years. Anthropometric measurements, fasting metabolic and hormonal profiles were obtained, and paired serum and cervical mucus samples collected on midluteal phase of the menstrual cycle were analyzed for DHA concentrations using ELISA. **Results**: Serum DHA levels were significantly higher in the PCOS group compared with controls (304.50 [167.75–593.00] vs. 168.50 [105.25–312.75] ng/L; median difference of 136.0 ng/L [95% CI: 18.46–284.50]; *p* = 0.015). Cervical mucus DHA levels tended to be lower in the PCOS group (189.50 [168.75–240.25] vs. 220.00 [189.50–241.75] ng/L; median difference of −30.50 ng/L [95% CI: −63.01 to −12.00]; *p* = 0.098). The serum-to-cervical mucus DHA ratio was significantly higher in the PCOS group (1.65 [0.85–2.83] vs. 0.80 [0.52–1.93]; median difference of 0.85 [95% CI: 0.10–1.60]; *p* = 0.002). **Conclusions**: Women with PCOS exhibited significantly elevated serum DHA levels and serum to cervical mucus DHA ratios compared to healthy controls, while cervical mucus DHA levels were similar between groups. The higher serum DHA and comparatively lower cervical mucus DHA in PCOS patients may indicate impaired DHA metabolism, slower metabolic processing, or reduced utilization of its active mediators. To our knowledge, this is the first study to examine DHA levels in both serum and cervical mucus in PCOS, highlighting the need for further large-scale studies.

## 1. Introduction

Polycystic ovary syndrome (PCOS) is a common endocrine disorder that has been recognized for decades, yet its etiology and pathogenesis remain incompletely understood. The prevalence of PCOS varies across populations and is estimated to range from 4% to 21% among women of reproductive age [1]. It is characterized as a chronic, multifactorial, polygenic, and familial condition, presenting with a heterogeneous spectrum of clinical manifestations that often necessitate a multidisciplinary management approach. The variability in symptom presentation underscores the need for clearly defined diagnostic criteria, which are essential not only for addressing short-term clinical outcomes but also for evaluating long-term health risks associated with the syndrome.

Chronic low-grade inflammation has emerged as a key factor in the pathophysiology of many long-term diseases. In PCOS, a proinflammatory state has been linked to insulin resistance, hyperandrogenism, and metabolic disturbances [2]. Dietary components, particularly bioactive lipids, play a critical role in modulating inflammatory processes. Certain polyunsaturated fatty acids (PUFAs), which are essential for energy metabolism and various physiological functions, cannot be synthesized endogenously and must be obtained from the diet. Among these, omega-3 fatty acids, specifically eicosapentaenoic acid (EPA) and docosahexaenoic acid (DHA), have been shown to exert anti-inflammatory, anti-thrombotic, and anti-arrhythmic effects, reduce serum triglyceride and LDL cholesterol levels, and improve immune function. Deficiencies in omega-3 fatty acids can manifest as fatigue, musculoskeletal pain, and increased susceptibility to infections [3,4]. Moreover, diets rich in omega-3 fatty acids have been reported to positively influence epigenetic mechanisms.

The pathophysiology of PCOS is further complicated by hyperinsulinemia, insulin resistance, and increased abdominal adiposity, regardless of obesity status. Excess visceral fat contributes to persistent low-grade systemic inflammation, reflected by elevated circulating inflammatory markers. During acute inflammatory responses, arachidonic acid (AA) and ω-6 PUFAs are metabolized by immune cells, and proinflammatory cytokines orchestrate a complex network of signaling pathways. Equally important as the initiation of inflammation is its self-limitation through active resolution processes. Disruption of these resolution mechanisms can result in sustained chronic inflammation, a hallmark of PCOS and other chronic diseases [5,6,7,8].

Active resolution of inflammation involves the conversion of alpha-linolenic acid (ALA) to ω-3 PUFAs (EPA and DHA), which are further metabolized into specialized pro-resolving mediators (SPMs). Cyclooxygenase (COX) and lipoxygenase (LOX) enzymes are integral to this process. SPM precursors, such as 18-HpETE, 17-HpDHA, and 14-HpDHA, are converted into terminal SPM families, including the E-series (RvE1, RvE2, RvE3; derived from EPA), D-series (RvD1, RvD2, RvD3, RvD4; derived from DHA), protectins, and maresins (derived from DHA) [9,10]. SPMs play a critical role in attenuating proinflammatory cytokine levels; reductions in SPM concentrations can lead to elevated inflammatory mediators, contributing to the chronic inflammatory state observed in PCOS.

Previous studies in PCOS patients have demonstrated that proinflammatory cytokines, such as tumor necrosis factor-alpha (TNF-α), are produced by activated macrophages and participate in apoptotic signaling pathways. Supplementation with DHA and/or EPA has been shown to inhibit TNF-mediated cytotoxicity by modulating arachidonic acid metabolism [11]. Animal studies further support the beneficial effects of ω-3 fatty acids on PCOS-related hormonal dysregulation, demonstrating improvements in FSH and testosterone levels following supplementation [12,13,14].

Despite these insights, there remains a notable gap in the literature; no studies to date have simultaneously assessed DHA concentrations in both serum and cervical mucus in patients with PCOS. The present study aims to address this gap by evaluating and comparing DHA levels in these biological compartments between PCOS patients and healthy controls. By elucidating the distribution of DHA in serum and cervical mucus, this research may contribute to a better understanding of PCOS pathophysiology and inform potential therapeutic strategies.

## 2. Materials and Methods

### 2.1. Patient Selection

This cross-sectional study was conducted at the Department of Obstetrics and Gynecology, Recep Tayyip Erdogan University Education and Research Hospital, in the outpatient gynecology clinic after ethics committee approval of the same university. Diagnosis of polycystic ovary syndrome (PCOS) was established according to the Rotterdam criteria [9]. Patients in the PCOS and control groups were matched for age and body mass index (BMI) prior to inclusion in the study.

A priori power analysis was performed to determine the required sample size for detecting differences in DHA concentrations between groups using IBM SPSS Statistics (Version 26.0; IBM Corp., Armonk, NY, USA) and R software (Version 4.3.0; R Foundation for Statistical Computing, Vienna, Austria). An effect size of 0.7 was selected because detecting smaller effect sizes (e.g., 0.3–0.5) would have required substantially larger study groups, which was not feasible within the recruitment capacity and practical constraints of the study setting. Using an effect size of 0.7, a two-sided α of 0.05, and 80% power, the minimum required sample size was calculated as 34 participants per group. To account for potential dropouts, 42 women were ultimately enrolled in each group (84 in total), providing a final sample size capable of detecting a moderate-to-large between-group difference corresponding to an effect size of approximately 0.62. 

### 2.2. Inclusion and Exclusion Criteria

- Inclusion criteria for the PCOS patient group: Women aged between 18 and 40 years who had been diagnosed with polycystic ovary syndrome (PCOS) were included. The diagnosis of PCOS and phenotypes was established based on the 2003 Rotterdam ESHRE/ASRM diagnostic criteria, which rely on ultrasound findings, medical history, clinical features, laboratory parameters, and physical examination.
- Exclusion criteria: Women with a diagnosis or suspicion of any kind of malignancy, history of metformin usage, postmenopausal period, pregnancy, or oral contraceptive therapy within the past three months
- Control group: Women aged between 18 and 40 years who presented for routine check-ups, had regular menstrual cycles, no obstetric or gynecological pathology (such as myoma, endometrioma, etc.), no acute or chronic infections (including upper/lower respiratory tract infections, urinary tract infections, etc.), and who had not started any medical treatment were included as healthy, voluntary participants.

Detailed medical histories were obtained and physical examinations were performed for patients presenting to the Gynecology Outpatient Clinic for diagnostic and therapeutic purposes. All participants underwent pelvic transvaginal ultrasonography, anthropometric measurements, including body weight (kg), waist circumference (>88 cm), and hip circumference (cm), and modified Ferriman–Gallwey score (mFGS) were recorded. The Body Mass Index (BMI) (kg/m^2^) was calculated as the ratio of body weight to the square of height. The waist-to-hip ratio was also determined, and a value greater than 0.85 was considered an indicator of central obesity.

To confirm the diagnosis of polycystic ovary syndrome (PCOS) and evaluate potential metabolic and hormonal complications, all participants underwent biochemical and hormonal assessments after 10–12 h of fasting. Fasting plasma glucose was measured (impaired glucose tolerance: 100–125 mg/dL; overt hyperglycemia: ≥126 mg/dL), along with lipid profile (total cholesterol > 200 mg/dL; triglycerides > 150 mg/dL; HDL < 100 mg/dL), C-reactive protein (CRP), and beta-human chorionic gonadotropin (β-hCG). Basal hormone profiles, including luteinizing hormone (LH), follicle-stimulating hormone (FSH), and estrogen, were evaluated on days 3–5 of the menstrual cycle. Progesterone levels were assessed at midluteal phase, and androgens, including total testosterone, 17-hydroxyprogesterone (17-OHP), and dehydroepiandrosterone sulfate (DHEA-S), were measured on days 3–5 of the cycle. Thyroid-stimulating hormone (TSH) and prolactin levels were also determined.

At the midluteal phase of the menstrual cycle, simultaneous blood and cervical mucus samples were collected. Blood samples were drawn into yellow-top gel-containing tubes, centrifuged, and the resulting sera, along with cervical mucus samples, were stored at −80 °C until analysis. Once the target number of patient and control samples was reached, specimens were gradually thawed, and docosahexaenoic acid (DHA) levels were quantified using ELISA kits (Invitrogen, Thermo Fisher Scientific, Waltham, MA, USA).

The ELISA kit and consumables used for DHA measurement in this study were provided by the Scientific Research Projects Unit of Recep Tayyip Erdogan University (TTU-2022-1453).

The study was reviewed and conducted in accordance with the Declaration of Helsinki and the Good Clinical Practice Guidelines, and it was reported that all procedures were performed in full compliance with these standards.

### 2.3. Statistical Analysis

Statistical analyses were performed using IBM SPSS Statistics (Version 26.0; IBM Corp., Armonk, NY, USA) and R software (Version 4.3.0; R Foundation for Statistical Computing, Vienna, Austria). Normality was assessed using the Kolmogorov–Smirnov test and visual inspection of histograms and Q–Q plots. Descriptive statistics (mean, standard deviation, median, 25th and 75th percentiles, frequency, and percentage) were used to summarize the data. Comparisons between PCOS and control groups were performed using Welch’s *t*-test or the Wilcoxon rank-sum test for continuous variables, and Pearson’s chi-squared test or Fisher’s exact test for categorical variables. Two-tailed statistical significance was set at *p* < 0.05.

Because serum and cervical mucus DHA concentrations demonstrated non-normal distributions, within-group associations were evaluated using Spearman’s rank correlation coefficient. To examine whether any potential relationship persisted after adjustment, a partial Spearman analysis was performed incorporating relevant clinical, hormonal, metabolic, and anthropometric covariates, including age, BMI, reproductive history (gravida, parity, abortions), mFGS score, FSH, LH, prolactin, TSH, total testosterone, DHEA-S, lipid parameters (total cholesterol, LDL, HDL, triglyceride), fasting glucose, waist and hip circumferences, and CRP.

Comparisons of serum DHA, cervical mucus DHA, and CRP levels across PCOS phenotype categories (A, C, and D) and the control group were conducted using the Kruskal–Wallis test due to non-normal data distributions. When a significant global difference was identified, pairwise post hoc analyses with Bonferroni correction were applied.

## 3. Results

Data from a total of 84 participants included in the study were analyzed. Comparison of baseline demographic characteristics between the groups revealed that age, body mass index (BMI), parity, number of living children, history of abortion, mFGS, and complaints of clinical oligomenorrhea or amenorrhea were significantly higher in the PCOS group (*p* < 0.05).

Comparison of baseline hormonal parameters demonstrated that mid-luteal progesterone, luteinizing hormone (LH), total testosterone (TT), and 17-hydroxyprogesterone (17-OH progesterone) were significantly elevated in the PCOS group (*p* < 0.05).

When lipid profiles and anthropometric measurements were compared between the two groups, waist circumference and waist-to-hip ratio were significantly higher in the PCOS group (*p* < 0.05). No significant differences were observed in hip circumference, total cholesterol, high-density lipoprotein (HDL), triglycerides, low-density lipoprotein (LDL), or fasting plasma glucose (FPG) between the groups (*p* > 0.05) (Table 1).

The serum-to-cervical mucus DHA ratio in the PCOS group was calculated as 361.95/197.55 = 1.83, whereas in the control group, it was 268.12/221.83 = 1.2 (*p* < 0.01).

The mean serum DHA levels were significantly higher in the PCOS group compared to the control group (*p* < 0.05). In contrast, cervical mucus DHA levels and serum CRP levels were comparable between the two groups (*p* > 0.05) (Table 2).

**Figure 1:**
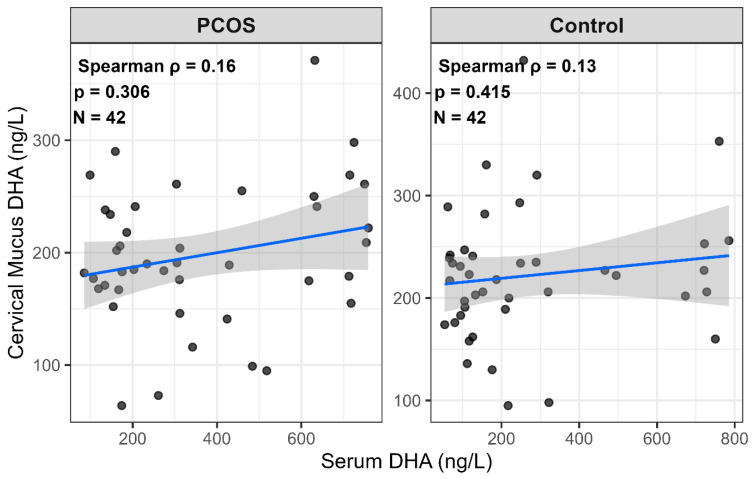
Scatterplots of the relationship between serum and cervical mucus DHA concentrations in PCOS and control groups [Scatterplots depicting the association between serum DHA and cervical mucus DHA concentrations in women with PCOS and healthy controls. Each panel includes the fitted linear regression line with its 95% confidence interval (grey shading). Spearman’s rank correlation coefficient (ρ), corresponding *p*-value, and sample size (n) are shown within each panel. No significant within-group correlation was observed in either group].

Unadjusted within-group analyses did not identify a significant correlation between serum and cervical mucus DHA concentrations. The associations were weak and non-significant in both groups (PCOS: Spearman’s ρ = 0.16, *p* = 0.31; controls: ρ = 0.13, *p* = 0.41). The corresponding scatterplots and regression fits are shown in Figure 1.

After adjustment for a broad panel of clinical, hormonal, metabolic, and anthropometric covariates (age, BMI, gravida, parity, number of abortions, mFGS score, FSH, LH, prolactin, TSH, total testosterone, DHEA-S, lipid profile, fasting glucose, waist and hip circumferences, and CRP), the within-group correlations remained weak and statistically non-significant (PCOS: partial ρ = 0.161, *p* = 0.475; controls: partial ρ = −0.072, *p* = 0.755). These adjusted results confirm that the lack of association between serum and cervical mucus DHA levels is not attributable to confounding by the evaluated covariates.

Serum DHA concentrations and the serum-to-cervical mucus DHA ratio differed significantly across PCOS phenotypes and the control group in global comparison (*p* = 0.023 and *p* = 0.014, respectively), whereas cervical mucus DHA (*p* = 0.073) and CRP levels (*p* = 0.252) did not show significant differences (Table 3). Although serum DHA levels tended to be higher in phenotypes A and C compared with phenotype D and controls, post hoc pairwise comparisons did not identify statistically significant differences between any individual phenotype pairs. In contrast, pairwise analyses of the serum-to-cervical mucus DHA ratio demonstrated a significantly higher ratio in phenotype A compared with controls (*p* = 0.040), with a similar but non-significant trend observed for phenotype C versus controls (*p* = 0.098). No additional phenotype-specific pairwise differences were identified.

## 4. Discussion

Docosahexaenoic acid (DHA) is one of the essential ω-3 PUFAs that must be obtained through diet and exerts significant beneficial effects on the cardiovascular system, lipid profile, neurological functions, and metabolic processes, in addition to its anti-inflammatory properties. However, for DHA to exert physiological efficacy, adequate intake alone is insufficient; proper functioning of metabolic pathways is also required. Epigenetic or enzymatic impairment within these pathways may lead to impaired DHA utilization, resulting in its biological inactivation and insufficient tissue concentrations.

In this study, PCOS was found to be associated with several metabolic alterations. Notably, serum DHA concentrations in PCOS patients were significantly higher than those in the control group, whereas DHA levels in cervical lavage fluid did not differ between groups. These findings may indicate alterations in DHA metabolism in PCOS.

Obesity is one of the most common comorbidities of PCOS. Visceral adiposity and central obesity are frequently observed in PCOS and are closely linked to insulin resistance. The literature reports that weight gain contributes to the development of PCOS, whereas PCOS adversely affects weight-loss capacity [15]. Alvarez-Blasco et al. reported a PCOS prevalence of 28% among overweight and obese women [16], and Ollila MM et al. demonstrated that BMI, particularly in early adulthood, is associated with PCOS [17]. Consistent with these findings, BMI, waist circumference, and waist-to-hip ratio differed significantly between the two groups in our study.

Regarding glucose metabolism, Tasali et al. reported increased risk of early impaired glucose tolerance and type 2 diabetes in PCOS [18], and Moran et al. found a higher prevalence of impaired glucose tolerance and type 2 diabetes [19]. In contrast, fasting glucose levels did not differ significantly between groups in our study.

Dyslipidemia is another well-recognized metabolic component of PCOS. Wild et al. reported that dyslipidemia is common in PCOS and may occur independently of BMI, with higher triglyceride, LDL-cholesterol, and non-HDL-cholesterol levels, and lower HDL-cholesterol levels, contributing to increased cardiovascular risk and warranting routine lipid screening [20]. Makhija et al. similarly detected significant alterations in triglycerides and HDL in PCOS [21]. However, in our study, no significant differences in total cholesterol, triglycerides, LDL, or HDL were observed. Regional dietary habits (fish consumption, tea, cabbage, etc.) may contribute to these differences.

Chronic inflammation plays a central role in the pathophysiology of many chronic disorders, including PCOS. Considering the association between PCOS—a proinflammatory condition—and DHA, it is well recognized that DHA and its metabolites are actively involved in the resolution of chronic inflammation. When an acute inflammatory response fails to self-resolve, the condition becomes chronic, and DHA, along with its intermediate metabolites, cannot adequately exert anti-inflammatory effects.

A meta-analysis investigating the association between PCOS and CRP demonstrated that CRP levels are elevated in PCOS independent of obesity, supporting the role of chronic low-grade inflammation in PCOS pathogenesis [22]. Oxidative stress, another inflammatory marker in PCOS, has also been examined for its relationship with endothelial dysfunction. Independent of obesity, mononuclear-cell-derived oxidative stress in PCOS may contribute to hyperandrogenism and insulin resistance [23]. Another study reported increased susceptibility to oxidative-stress-induced DNA damage in PCOS, with free testosterone correlating with DNA susceptibility [24]. A meta-analysis assessing interactions among hormones, oxidative stress, and inflammatory factors revealed that ω-3 PUFA supplementation improved antioxidant capacity as well as CRP, SHBG, LH, and testosterone levels, but had no significant influence on DHEA-S, FSH, or other androgen levels [25]. In a 2014 study, Ouladsahebmadarek et al. found that rats with induced PCO receiving ω-3 supplementation and lower-carbohydrate diets exhibited increased antioxidant and FSH levels and decreased testosterone [13].

In another experimental study, following PCO induction, a DHA-induced PCOS model in rats was used to investigate the effects of various ω-3 fatty acids (synthetic ω-3 containing ALA, EPA, DHA; flaxseed oil; and fish oil) on lipid profiles. Significant reductions in cholesterol, triglycerides, LDL, blood glucose, testosterone, LH, and insulin were observed, alongside increases in HDL [14], supporting the beneficial effects of ω-3 fatty acids on lipid and hormonal regulation in PCOS.

A review examining the inflammatory effects of EPA and DHA reported that DHA has anti-inflammatory properties, while the effects of EPA remain uncertain. Age was identified as the most important determinant, with stronger adipocyte responses in young compared with mature or aged adipocytes [26]. Another study suggested that a global deficiency of DHA and EPA may contribute to increased cardiovascular mortality risk [27]. A meta-analysis further demonstrated that ω-3 fatty acids may benefit insulin resistance, hypercholesterolemia, and hypertriglyceridemia in PCOS [28]. Some researchers argue, however, that these effects may be dose-dependent or limited by insufficient production of active DHA-derived mediators, suggesting the need for supplementation with active metabolites or higher DHA doses [9,29].

Moreover, evidence increasingly indicates that DHA’s biological effects depend not solely on circulating levels but also on the production of specialized pro-resolving mediators (SPMs), particularly 17-H-DHA and D-series resolvins, which are critical for inflammatory resolution. As reported by Neuhofer et al. (2013) [29], DHA’s anti-inflammatory potency is primarily mediated through these metabolites; thus, elevated circulating DHA may not confer expected benefits in individuals with impaired SPM production. Reduced levels of 17-H-DHA—a key intermediate and SPM precursor—have been associated with obesity-related inflammatory dysregulation. Supplementation with 17-H-DHA has been shown to dose-dependently reduce NFκB activation while increasing PPARγ and adiponectin expression, thereby exerting anti-inflammatory effects. Comparative analyses demonstrate that both DHA and its metabolite 17-H-DHA exhibit anti-inflammatory properties and enhance insulin sensitivity; however, 17-H-DHA more effectively improves glucose tolerance, reduces plasma insulin concentrations, and increases insulin responsiveness [29]. In addition, impaired DHA metabolism has been linked to diminished anti-inflammatory responses, particularly through reduced 5-LOX enzymatic activity, which lowers 17-OH-DHA levels and disrupts D-series resolvin synthesis. Decreased SPMs and their precursors thereby limit the anti-inflammatory potential of DHA [10]. This mechanistic framework may help explain the lack of difference in cervical mucus DHA concentrations despite elevated serum DHA levels observed in our study. Although serum DHA increased, impaired SPM production or limited tissue transport may have prevented effective anti-inflammatory activity in target tissues. This suggests that DHA supplementation in PCOS cannot be evaluated solely on the basis of serum levels and that higher doses or supplementation with active metabolites may be required.

The beneficial effects of ω-3 fatty acids have been demonstrated even in inflammatory processes such as atherosclerosis from fetal life onward [30,31]. Helland et al. reported higher cognitive scores in children whose mothers received fish oil supplementation [32]. Several studies have also shown that DHA deficiency may lead to neurodevelopmental impairment [3,4,30,33]. In 2000, Yano et al. demonstrated that fatty acid supplementation (DHA and/or EPA) reduced TNF-induced cytotoxicity by inhibiting arachidonic-acid metabolic pathways, thereby decreasing apoptosis [11].

Low SHBG levels—a characteristic feature of hyperandrogenism in PCOS—are strongly associated with insulin resistance. While several studies have reported beneficial metabolic effects of PUFA-rich diets [34], others have suggested that inflammation may contribute to hyperandrogenism [35]. Recent research has also shown that low DHA levels are associated with reduced SHBG and increased insulin resistance [36].

Current evidence on disrupted fatty-acid metabolism in PCOS supports the serum–tissue dissociation observed in our study. Tian et al. (2023) demonstrated that serum fatty-acid profiles in PCOS women differ significantly from controls, independent of BMI and correlate strongly with metabolic risk markers, suggesting alterations not only in fatty-acid levels but also in their utilization and distribution [37]. Similarly, Lu et al. (2022) reported inverse correlations between serum EPA, DPA, DHA, and metabolic/hormonal abnormalities in PCOS, with higher DHA levels associated with lower BMI, fasting insulin, total testosterone, and hs-CRP, and higher SHBG and FSH [38]. Together, these findings suggest that elevated circulating DHA in PCOS may represent a compensatory response, whereas tissue transport and local biotransformation may be impaired by metabolic stress, inflammation, or hormonal imbalance. The lack of difference in cervical mucus DHA levels in our study, despite elevated serum DHA in PCOS patients, aligns with this mechanism. This supports the notion that DHA may not adequately reach target tissues, that serum concentrations alone are insufficient to predict biological activity, and that DHA bioavailability may be limited at the tissue level in PCOS. Accordingly, elevated serum DHA may reflect impaired tissue uptake rather than a protective adaptation.

Our literature search found no prior studies investigating DHA levels in blood or cervical mucus in PCOS. In the present study, DHA concentrations in serum and cervical mucus were compared between PCOS patients and healthy controls. While serum DHA differed significantly between groups, cervical mucus DHA did not.

Differences between dietary intake and circulating ω-3 fatty-acid levels may arise due to metabolic alterations both in the general population and in PCOS. Genetic factors may also influence DHA metabolism. Any impairment within these pathways may disrupt DHA absorption and metabolism, diminishing its physiological actions and limiting bioavailability. Consequently, metabolic blockade or impairment in DHA metabolism in PCOS may obscure its expected clinical effects. In this context, dietary requirements for DHA or its active metabolites may be higher to achieve adequate circulating concentrations and ensure physiological effectiveness.

Given the essential dietary requirement for DHA and the observed discrepancies between serum and cervical mucus levels, impaired tissue distribution (e.g., reduced cervical transfer) or metabolic blockade may result in reduced tissue DHA concentrations. Thus, PCOS patients may fail to exhibit the expected cardiometabolic and anti-inflammatory benefits of DHA, suggesting that higher supplementation doses or direct administration of active mediators may be necessary.

In our study, while anthropometric parameters (BMI and waist-hip ratio) and hormonal profiles were consistent with PCOS, CRP and lipid-profile parameters were similar between groups, possibly reflecting regional dietary habits (fish, tea, kale, etc.). Based on our statistical analyses, significantly elevated serum DHA levels in PCOS, combined with unchanged cervical-mucus levels and a lower serum/cervical ratio, may provide a plausible explanation for these findings.

## 5. Conclusions

In conclusion, serum DHA levels and serum to cervical mucus DHA ratio were found to be significantly higher in patients with PCOS compared to the control group, whereas DHA levels in cervical lavage fluid were similar between the two groups. The observation of elevated serum DHA levels and relatively lower DHA concentrations in cervical mucus (as indicated by the serum-to-cervical mucus ratio in PCOS patients compared to controls) may suggest that the increased DHA in PCOS patients is due to impaired metabolism, slower metabolic processing, or an inability of the body to effectively utilize its active mediators. To our knowledge, this is the first study in the literature to investigate this topic, and further large-scale studies are warranted to confirm and expand upon these findings.

## Figures and Tables

**Table 1:** Comparison of baseline demographic characteristics, hormonal parameters, lipid profiles, and anthropometric measurements.

Parameter	PCOS Group (n = 42)	Control Group (n = 42)	Effect Size	*p*-Value
Age (years)	28.00 (26.00–30.00)	31.00 (27.00–34.00)	−3.00 (−6.35, 0.35)	0.024 *
BMI (kg/m^2^)	27.14 (23.41–31.23)	25.27 (22.01–27.77)	1.87 (−0.78, 4.52)	0.043 *
Gravida (n)	0.00 (0.00–1.00)	1.00 (0.00–2.00)	−1.00 (−2.08, 0.08)	0.062 ^†^
Parity (n)	0.00 (0.00–1.00)	1.00 (0.00–2.00)	−1.00 (−1.68, −0.32)	0.005 **
Abortions (n)	0.00 (0.00–0.00)	0.00 (0.00–0.00)	0.00 (0.00, 0.00)	0.036 *
mFGS	14.50 (10.50–19.00)	0.00 (0.00–0.00)	14.50 (11.52, 17.48)	<0.001 ***
Oligo-/amenorrhea n (%)	36 (85.7%)	0 (0%)	-	0.000 *
Smoking n (%)	10 (23.8%)	7 (16.7%)	1.56 (0.53, 4.59)	0.417
Mid-luteal Progesterone (ng/mL)	0.82 (0.57–4.54)	8.75 (1.75–13.44)	−7.93 (−10.47, −5.39)	<0.001 ***
FSH (mIU/mL)	5.39 (4.39–6.50)	5.57 (4.66–6.98)	−0.18 (−1.02, 0.66)	0.426
LH (mIU/mL)	7.10 (5.16–8.46)	5.00 (3.88–5.85)	2.09 (0.81, 3.38)	<0.001 ***
Estradiol (pg/mL)	44.47 (38.12–55.60)	51.55 (41.40–69.11)	−7.09 (−15.53, 1.36)	0.106
PRL (ng/mL)	10.26 (7.96–12.45)	9.09 (7.27–13.23)	1.17 (−1.28, 3.63)	0.724
TSH (µIU/mL)	2.41 ± 1.25	2.38 ± 1.06	0.03 (−0.48, 0.53)	0.912
Total Testosterone (ng/dL)	30.49 (9.32)	22.87 (7.45)	7.62 (3.93, 11.31)	<0.001 ***
DHEA-S (µg/dL)	226.44 ± 103.22	190.18 ± 98.99		0.104
17-OH Progesterone (ng/mL)	1.26 ± 0.52	0.81 ± 0.41	0.44 (0.24, 0.65)	<0.001 ***
Total Cholesterol (mg/dL)	196.6 ± 34.1	185.4 ± 27.7	11.14 (−2.35, 24.64)	0.104
HDL (mg/dL)	48.50 (40.17–53.75)	51.00 (45.00–59.75)	−2.50 (−8.57, 3.57)	0.125
Triglycerides (mg/dL)	105.00 (76.25–134.00)	88.50 (74.00–107.75)	16.50 (−2.32, 35.32)	0.273
LDL (mg/dL)	123.59 ± 31.16	112.12 ± 27.01	11.47 (−1.19, 24.13)	0.075 ^†^
Fasting Blood Glucose (mg/dL)	86.50 (81.50–93.25)	87.50 (82.50–93.00)	−1.00 (−4.67, 2.67)	0.694
Waist Circumference (cm)	86.00 (77.25–95.50)	79.00 (75.00–88.00)	7.00 (1.06, 12.94)	0.019 *
Hip Circumference (cm)	106.50 (100.25–115.00)	102.00 (98.00–110.00)	4.50 (−1.38, 10.38)	0.058 ^†^
High Waist-to-Hip Ratio	11 (26.19%)	4 (9.52%)	0.30 (0.09, 1.02)	0.055 ^†^
Serum DHA (ng/L)	304.50 (167.75–593.00)	168.50 (105.25–312.75)	136.00 (18.46, 284.50)	0.015 *
Cervical Mucus DHA (ng/L)	189.50 (168.75–240.25)	220.00 (189.50–241.75)	−30.50 (−63.01, −12.00)	0.098 ^†^
CRP (mg/L)	3.25 (2.82–5.50)	3.15 (2.62–3.80)	0.10 (−0.90, 0.60)	0.108

PCOS: polycystic ovary syndrome; BMI: body mass index; mFGS: modified Ferriman–Gallwey score; FSH: follicle-stimulating hormone; LH: luteinizing hormone; PRL: prolactin; TSH: thyroid-stimulating hormone; DHEA-S: dehydroepiandrosterone sulfate; HDL: high-density lipoprotein; LDL: low-density lipoprotein; DHA: docosahexaenoic acid; CRP: C-reactive protein; n, number. Numeric data are shown as mean ± SD or median [25th, 75th percentile] based on normality, and categorical data as n/N (%). Effect sizes are given as mean difference (95% CI) for normally distributed data, median difference (95% CI) for non-normally distributed data, and OR [95% CI] for categorical variables, with the control group as the reference. Welch’s *t*-test, Wilcoxon rank-sum test, and Pearson’s chi-squared test or Fisher’s exact test were used as appropriate. Significance indicators: * *p* < 0.05, ** *p* < 0.01, *** *p* < 0.001, ^†^ *p* < 0.10 (trend).

**Table 2:** Comparison of CRP and DHA levels.

Parameter	PCOS Group (n = 42)	Control Group (n = 42)	Effect Size	*p*-Value
Serum DHA (ng/L)	304.50 (167.75–593.00)	168.50 (105.25–312.75)	136.00 (18.46, 284.50)	0.015 *
Cervical Mucus DHA (ng/L)	189.50 (168.75–240.25)	220.00 (189.50–241.75)	−30.50 (−63.01, −12.00)	0.098 ^†^
Serum-to-cervical mucus DHA ratio	1.65 (0.85–2.83)	0.80 (0.52–1.93)	0.85 (0.10, 1.60)	0.002 **
CRP (mg/L)	3.25 (2.82–5.50)	3.15 (2.62–3.80)	0.10 (−0.90, 0.60)	0.108

PCOS: polycystic ovary syndrome; DHA: docosahexaenoic acid; CRP: C-reactive protein; n, number. Numeric data are shown as median [25th, 75th percentile]. Effect sizes are given as median difference (95% CI) with the control group as the reference. The Wilcoxon rank-sum test was used. Significance indicators: * *p* < 0.05, ** *p* < 0.01, ^†^ *p* < 0.10 (trend).

**Table 3:** Comparison of CRP and DHA levels according to PCOS phenotypes.

	PCOS Phenotype A (n = 42)	PCOS Phenotype C (n = 6)	PCOS Phenotype D (n = 3)	Control (n = 42)	*p*-Value
Serum DHA (ng/L)	311 (167–618)	395 (274–637)	154 (119–174)	169 (105–320)	0.023 *
Cervical Mucus DHA (ng/L)	202 (175–241)	188 (184–241)	152 (64–168)	220 (189–242)	0.073 ^†^
Serum-to-cervical mucus DHA ratio	1.70 [0.83–2.95]	2.12 [1.49–2.87]	1.01 [0.71–2.72]	0.80 [0.52–2.05]	0.014 *
CRP (mg/L)	3.60 (2.90–5.70)	2.95 (2.70–3.70)	3.00 (2.60–7.00)	3.15 (2.60–3.80)	0.252

PCOS: polycystic ovary syndrome; DHA: docosahexaenoic acid; CRP: C-reactive protein; n, number. Numeric data are presented as median [25th–75th percentile]. The Kruskal–Wallis test was used because DHA and CRP levels did not follow a normal distribution. Significance indicators: * *p* < 0.05, ^†^ *p* < 0.10 (trend).

## Data Availability

All data supporting the results of this study are available from the corresponding author upon reasonable request.

## Abbreviations

17-α OHP	17-Alpha Hydroxyprogesterone
ASRM	American Society for Reproductive Medicine
BMI	Body Mass Index
CRP	C-Reactive Protein
DHA	Docosahexaenoic Acid
DHEA-S	Dihydroepiandrostenedione Sulfate
E_2_	Estradiol
EPA	Eicosapentaenoic
ESHRE	European Society of Human Reproduction and Embryology
FPG	Fasting Plasma Glucose
FSH	Follicle-Stimulating Hormone
fT	Free Testosterone
hCG	Human Chorionic Gonadotropin
HDL	High-Density Lipoprotein
IL-1β	Including Interleukin-1β
IL-6	Interleukin-6
LDL	Low-Density Protein
LH	Luteinizing Hormone
LOX	Lipoxygenase
mFGS	modified Ferriman Gallwey Score
PCOS	Polycystic Ovary Syndrome
PRL	Prolactin
SPM	Specialized Pro-Resolving Mediators
SPSS	Statistical Package for the Social Sciences
TNF-α	Tumor Necrosis Factor-Alpha
TSH	Thyroid-Stimulating Hormone
TT	Total Testosterone
